# Human umbilical cord mesenchymal stem cells facilitate the up‐regulation of miR‐153‐3p, whereby attenuating MGO‐induced peritoneal fibrosis in rats

**DOI:** 10.1111/jcmm.13622

**Published:** 2018-04-14

**Authors:** Dong Li, Zhenyu Lu, Xiyuan Li, Zhongwei Xu, Jianqing Jiang, Zhenfeng Zheng, Junya Jia, Shan Lin, Tiekun Yan

**Affiliations:** ^1^ Department of Nephrology General Hospital of Tianjin Medical University Tianjin China; ^2^ Tianjin Precell Biotechnology Co., Ltd. Huayuan Industrial District Tianjin China; ^3^ Precision Medical Center General Hospital of Tianjin Medical University Tianjin China; ^4^ Central Laboratory Logistics University of the Chinese People's Armed Police Force Tianjin China

**Keywords:** epithelial to mesenchymal transition, human umbilical cord mesenchymal stem cells, microRNA, peritoneal fibrosis, peritoneal mesothelial cells

## Abstract

MiRNAs contribute greatly to epithelial to mesenchymal transition (EMT) of peritoneal mesothelial cells (PMCs), which is a crucial step in peritoneal fibrosis (PF). In this study, we tried to profile whether miRNA expression differences exist after human umbilical cord mesenchymal stem cells (hUCMSCs) treatment in PF rats and investigate the possible role of miR‐153‐3p involved in anti‐EMT process. We randomly assigned 34 rats into three groups: control group (Group Control), MGO‐induced PF rats (Group MGO) and hUCMSCs‐treated rats (Group MGO + hUCMSCs). MiRNA microarrays and real‐time PCR analyses were conducted in three groups. α‐SMA, Snail1 and E‐cadherin expression were detected by Western blot. Luciferase reporter assays were used to detect the effects of miR‐153‐3p overexpression on Snai1 in rat peritoneal mesothelial cells (RPMCs). We identified differentially expressed miRNAs related to EMT, in which miR‐153‐3p demonstrated the greatest increase in Group MGO + hUCMSCs. Transient cotransfection of miR‐153‐3p mimics with luciferase expression plasmids resulted in a significant repression of Snai1 3′‐untranslated region luciferase activity in RPMCs. These studies suggest that miR‐153‐3p is a critical molecule in anti‐EMT effects of hUCMSCs in MGO‐induced PF rats. MiR‐153‐3p might exert its beneficial effect through directly targeting Snai1.

## 1 INTRODUCTION

Chronic kidney disease (CKD) represents a common health problem worldwide, and their prevalence continues to increase.^1^ Most patients with CKD were usually occult and, so far, lack of early diagnosis and treatment methods, which is eventually led to end‐stage renal disease (ESRD). As a treatment at home, peritoneal dialysis (PD) has become a common treatment approach for patients with ESRD. However, long‐term PD can lead to morphological and functional changes in the peritoneum, and subsequent fibrosis and angiogenesis.^2^, ^3^ Peritoneal fibrosis (PF), which is lack of effective prevention and control countermeasures, is one of the most important factors that force patients with ESRD to interrupt from long‐term PD, hence restricts the application and development of PD.^4^ How to delay or even block PF process has become an urgent issue in research field.

Mesenchymal stem cells (MSCs) from the early development of mesoderm and ectoderm belong to the pluripotent stem cells, which have been suggested as promising candidates for various cell‐based therapeutic approaches including treatments for PF.^5^ Human umbilical cord mesenchymal stem cells (hUCMSCs) are one of the typical MSCs, which have advantages such as low immunogenicity, painless collection procedure, faster expansion in vitro and ethical access compared to MSCs from other sources.^6^ Therefore, the administration of hUCMSCs might be considered as a potential strategy for the intervention point of PF. Despite this potential, the mechanisms and signalling pathways involved in hUCMSCs‐mediated PF repair are poorly understood.

MicroRNAs (miRNAs) are a group of endogenous, non‐coding small RNAs that have recently been implicated in the regulation of gene expression on the post‐transcriptional level in multiple biological processes.^7^ Recently, several reports have demonstrated the association between miRNAs and PF, which indicated the emerging roles of miRNAs in the pathogenesis of PF.^8^, ^9^ However, further studies are needed to determine whether and which miRNA is involved in hUCMSCs‐mediated PF healing in rats.

In this study, we tested the protective effects of hUCMSCs treatment on methylglyoxal (MGO)‐induced PF in rats and tried to profile whether differences existed in peritoneal miRNA expression of control rats, MGO‐induced PF rats and hUCMSCs‐treated rats. We hypothesized that the greatest increased miR‐153 is overlapped among the three groups contributed to the attenuation of MGO‐induced PF in rats.

## 2 MATERIALS AND METHODS

### 2.1 Animals

A total of 34 male Wistar rats, 180‐220 g body weight, were purchased from Center of Radiation Medicine of Peking Union Medical College. The peritoneal dialysate fluid (PDF) with 20 mmol/L MGO was prepared by adding MGO to 2.5% PDF (Baxter, Guangzhou, China) and free of endotoxin for in vivo delivery. Twenty‐four rats were injected intraperitoneally daily with 100 mL/kg PDF with 20 mmol/L MGO. The rest rats, which also underwent intraperitoneal injection, were prepared as control treated with normal saline (Group Control, n* *=* *10). Two weeks after injection, intraperitoneal‐injected rats were randomly divided into two groups. One group received 2 × 10^6^ hUCMSCs infusion via a tail vein only one time to intervent PF (Group MGO + hUCMSCs, n* *=* *12). Another group was given only normal saline instead of hUCMSCs (Group MGO, n* *=* *12). Two weeks after hUCMSCs administration, rats were anaesthetized by intraperitoneal injection with sodium pentobarbital 50 mg/kg body weight. After that, 20 mL of 2.5% PDF was intraperitoneally injected and kept in peritoneal cavity for 2 hour. All dialysate in the peritoneal cavity was carefully collected through a midline incision. Blood sample was obtained by a direct cardiac puncture. Plasma creatinine concentration (P), dialysate creatinine concentration (D), dialysate fluid glucose concentration (D2) and PDF glucose concentration (D0) were determined by automatic biochemical analyser (HITACHI 7170A, Japan). Ultrafiltration volume (UF) was counted as followed: UF = the resulting dialysate (mL) ‐20(mL). The peritoneum of individual rats was also sampled for histological analysis, gene expression analysis and miRNA microarray analysis. All procedures were carried out in accordance with the approval of the ethics committee of Tianjin Medical University.

### 2.2 Isolation, cultivation, expansion, differentiation and identification of hUCMSCs

hUCMSCs were isolated and cultured with the tissue block attachment method as described previously.^10^ Fifteen fresh human umbilical cords were obtained from healthy mothers at Tianjin Central Hospital of Gynecology Obstetrics following their informed consent, and the experimental procedures were approved by the ethics committee of Tianjin Medical University. hUCMSCs were cultured for expansion in DMEM/F12 supplemented with 10% FBS and 100 U/mL penicillin/streptomycin, at 37°C in a 5% CO_2_ incubator. hUCMSCs between the fourth and sixth passages were collected for cell infusion. hUCMSCs (the 6th passages, 1 × 10^6^) were stained with anti‐CD34, CD45, CD90 and CD105 antibodies conducted at room temperature for 30 minutes in dark (1:500; BD Pharmingen, San Diego, CA, USA). Flow cytometry (Gilson, Middleton, WI, USA) was used to analyse antibody binding. Multipotency of hUCMSCs was confirmed by osteogenic and adipogenic differentiation.

### 2.3 Histological and immunohistochemical analysis

Peritoneum was removed from rats and fixed in 10% phosphate‐buffered formalin. The fixed tissue was then embedded in paraffin at 60°C and cut transversely into 2‐ to 3‐μm‐thick sections. Some sections were stained with haematoxylin and eosin (HE) to analyse cell type and identify collagen fibres with Sirius Red histochemistry. Peritoneal thickness was measured using image analysis software Image‐Pro Plus 5.0. Thickness was measured at 30 points per site (at 0.5‐mm intervals within a range of 1.5 cm), and the average was calculated. The other sections were pre‐incubated with blocking solution with bovine serum albumin (Zhongshan Goldenbridge, Beijing, China) for 10 minutes. Staining was performed by avidin‐biotin‐peroxidase complex technique according to manufacturer's protocol. The slides were incubated with primary antibodies, rabbit anti‐rat α‐SMA polyclonal antibody (1:400, Santa Cruz Biotechnology), goat anti‐rat Snail1 monoclonal antibody (1:400, Santa Cruz Biotechnology), rabbit anti‐rat E‐cadherin monoclonal antibody (1:400, Epitomics) and rabbit anti‐rat TGF‐β1 polyclonal antibody (1:200, Santa Cruz Biotechnology). Then, they were exposed overnight at room temperature and followed by a 40‐minute incubation with the secondary antibodies and PV‐6001 goat anti‐rabbit IgG‐HRP multimer (Zhongshan Goldenbridge, Beijing, China). Negative control sections were incubated with PBS instead of primary antibody. The areas of α‐SMA, Snail1, E‐cadherin and TGF‐β1 were assessed in predetermined fields that are captured by a digital camera. The image analyses of stained area were performed by Image‐Pro Plus 5.0 in 10 fields. The measurements were conducted by two investigators, independently.

### 2.4 MiRNA microarray analysis

Total RNAs were isolated from rat peritoneum of Group Control (n* *=* *3), Group MGO (n* *=* *3) and Group MGO + hUCMSCs (n* *=* *3) using mirVana™ miRNA Isolation Kit (Ambion, Austin, Texas, USA) according to the manufacturer's instructions. 5‐μg good quality RNAs were used for miRNA expression analysis, performed by TaqMan MiRNA Array v2.0 (Applied Biosystems, Foster City, CA, USA).

### 2.5 Quantitative reverse transcription‐polymerase chain reaction (qRT‐PCR)

Quantification of miRNAs expression levels was assessed via qRT‐PCR using specific TaqMan^®^ assays according to the instructions of the manufacturer (Applied Biosystems, Foster City, CA, USA). U6 RNA was used as normalizer.

### 2.6 Primary culture of RPMCs and grouping

Rat peritoneal mesothelial cells (RPMCs) were isolated and cultured as described previously.^11^ Briefly, 25 mL of 0.25% trypsinase and 0.02% EDTA‐Na_2_ were intraperitoneally infused into the abdominal cavities of individual rats. The abdominal fluid was collected under sterile conditions after 2 hours. Isolated RPMCs were harvested and grown in DMEM/F12 medium supplemented with 20% (v/v) FCS, at 37°C, in 5% CO_2_ culture conditions. Rat peritoneal mesothelial cells were confirmed by morphological polygonal cobblestone appearance and expression of the mesothelial‐specific marker cytokeratin‐2. Cells between passages 2 and 3 grown as a monolayer to 80% confluence were used for the following experiments. Rat peritoneal mesothelial cells were grown to confluence at the density of 1 × 10^5^ cells per well in a twelve‐well plate on the upper compartment of Transwell (Corning, Lowell, MA) and incubated for 24 hour (Group Normal RPMCs). Other RPMCs were grouped as follows: RPMCs stimulated by 2.5 ng/mL TGF‐β1 for 24 hour (Group RPMCs+TGF‐β1), RPMCs stimulated by 2.5 ng/mL TGF‐β1 and cocultured with hUCMSCs (1 × 10^5^cells, inoculated into the lower compartment) for 24 hour (Group RPMCs+TGF‐β1 + hUCMSCs), RPMCs stimulated by 2.5 ng/mL TGF‐β1 and treated with hUCMSCs‐conditioned medium (CM) (Group RPMCs + TGF‐β1 + hUCMSCs‐CM). MiR‐153‐3p was detected by real‐time PCR. MiR‐153‐3p was also detected in hUCMSCs with or without 2.5 ng/mL TGF‐β1 stimulation for 24 hour. (Group Normal hUCMSCs and Group hUCMSCs + TGF‐β1).

### 2.7 Preparation of CM

To study the influence of soluble factors produced by hUCMSCs, CM from the culture of hUCMSCs at passage 2‐4 was prepared. hUCMSCs in 80%‐90% confluent were washed three times with PBS and then fed with serum‐free medium for 24 hour. The medium was collected, and the cellular debris was removed through centrifugation at 671g for 10 minutes. This supernatant was subsequently used as hUCMSCs‐CM.

### 2.8 Western blot

Cells were lysed in hypotonic lysis buffer, and equal amounts of protein were loaded onto an 8% sodium dodecyl sulphate (SDS)‐polyacrylamide gel. Separated proteins were transferred into a nitrocellulose membrane and blocked with 8% non‐fat milk at room temperature for 1 hour. Membranes with proteins were incubated with rabbit anti‐rat α‐SMA polyclonal antibody (1:200, Santa Cruz Biotechnology), goat anti‐rat Snail1 monoclonal antibody (1:200, Santa Cruz Biotechnology) and rabbit anti‐rat E‐cadherin monoclonal antibody (1:400, Epitomics) overnight followed by incubation with secondary horseradish peroxidase antibodies according to the manufacturer's instructions. Enhanced chemiluminescence method was used to determine protein expression.

### 2.9 DNA construction

MiR‐153‐3p mimics and control mimics were synthesized by GenePharma (GenePharma, Shanghai, China). The wild‐type 3′ UTR fragment of rat Snai1 that contained putative binding site for miR‐153‐3p was amplified from mesothelial cells cDNA library and inserted into pcDNA3.1 plasmid. PCR primers sets of Snai1 were as follows: Forward, 5′‐CTACTGGACCCACCTTAGCATGTGTCCTAT‐3′; Reverse, 5′‐ACTTGCTACTCTGCCCTAAGGATCCTAAAGTTA‐3′. The oligonucleotide (300 to 400) of Snai1 3′ UTR was synthesized and inserted into pmirGLO luciferase plasmid at the XbaI‐digested site. Putative miR‐153‐3p binding site CUAUGCAA (nt 354‐361) was mutated into AUCCUGCA by oligonucleotide‐directed PCR (Figure 7B).

### 2.10 Liposome‐mediated plasmid transfection

MiR‐153‐3p mimics, control mimics, miR‐153‐3p negative control and anti‐miR‐153‐3p molecules were synthesized by GenePharma (GenePharma, Shanghai, China). MiR‐153‐3p mimics molecules and negative control miRNAs were transfected into RPMCs at a final concentration of 40 nmol/L by Lipofectamine 2000 (Invitrogen, Carlsbad, CA, USA). According to the Invitrogen manual, 6‐hole culture plate was used and culture medium containing 5 × 10^5^ cells was added to each hole at 37°C in 5% CO_2_ culture conditions to 70%‐80% confluence. After washing with OPTI‐MEM, 1 mL OPTI‐MEM medium was added without FBS. 200 μL plasmid‐liposome complexes (plasmid/liposome is 4 μg/10 μg) were added to the cells for incubation of 6 hour. After treatment, cells were cultured with 2 mL medium containing 10% FBS. The transfection efficiency was detected through real‐time PCR.

For miRNA targeting luciferase assay, RPMCs were cotransfected with luciferase vector, including the 3′ UTR of Snail, miR‐153‐3p mimics or miR‐153‐3p mimics negative control anti‐miR‐153‐3p molecules or anti‐miR‐153‐3p negative control at a final concentration of 40 nmol/L by Lipofectamine 2000 (Invitrogen, Carlsbad, CA, USA). Luciferase assays were performed by the dual luciferase reporter assay system (Promega, Madison, WI, USA) for 48 hour after transfection. Renilla luciferase activity was normalized to firefly luciferase expression for each sample (n = 3).

### 2.11 Statistical analysis

SPSS 16.0 software (SPSS Inc., Chicago, IL, USA) was used for the data processing. *P *<* *.05 was required for results to be considered statistically significant. Data were presented as Mean ± SEM and were compared by Student's *t* test or ANOVA followed by a post‐SNK *q* test as appropriate.

## 3 RESULTS

### 3.1 Characterization of cell cultures

**Figure 1:**
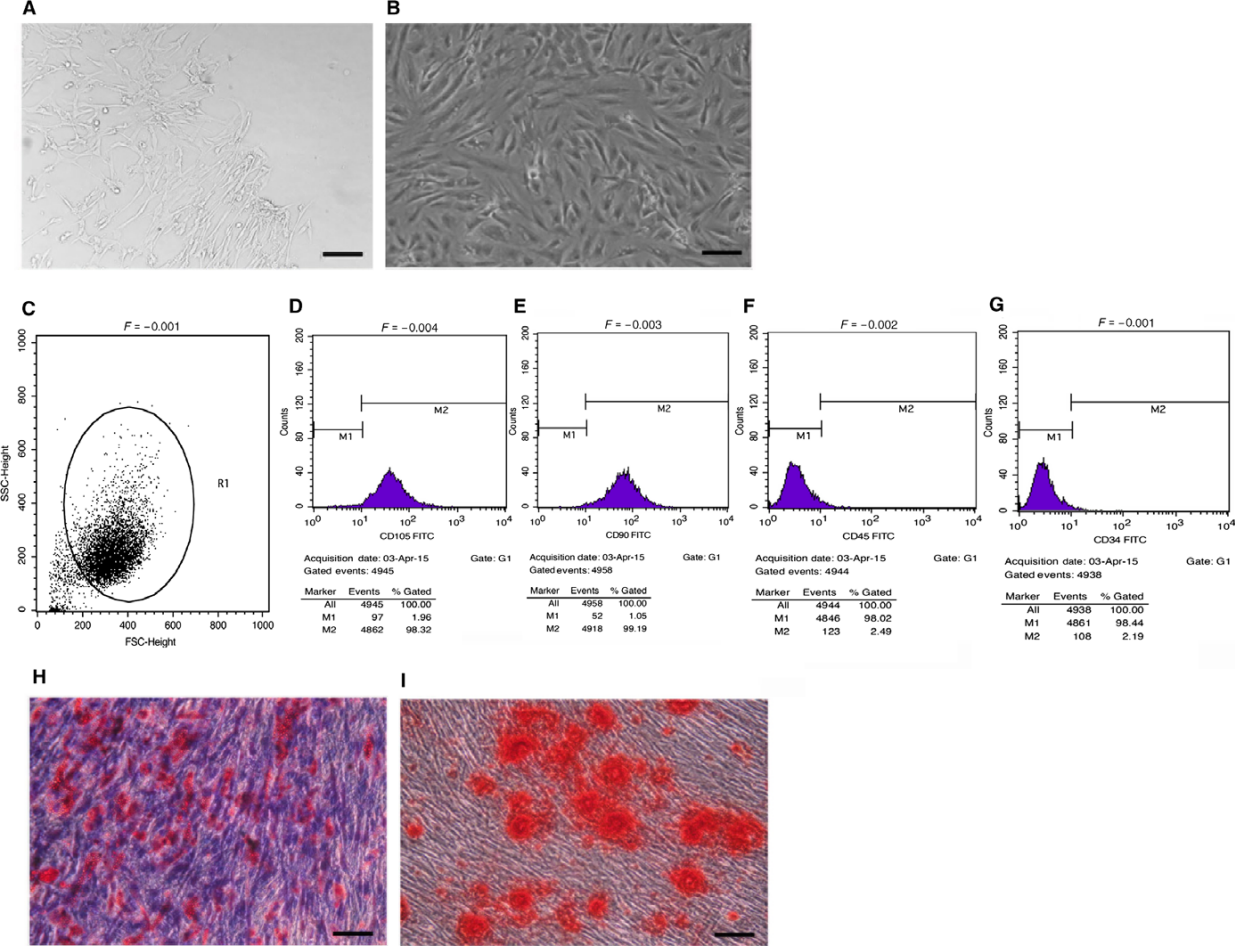
Biological characterization of hUCMSCs. A, Light microscopy image of hUCMSCs cultured for 14 days (×100 magnification, bar = 200 μm). B, Light microscopy image of hUCMSCs cultured for 20 days (×400 magnification, bar = 50 μm). C‐G, Expression of hUCMSCs surface markers. hUCMSCs had high expression of CD105 (C) and CD90 (D), but did not express CD34 (E) or CD45 (F). H, I, hUCMSCs multipotency determination. Adipogenic (H) and osteogenic (I) differentiations were determined using Oil Red O staining and Alizarin Red staining, respectively (×100 magnification, bar = 200 μm)

After 14 days in culture, scattered spindle‐shaped cells were found around the tissue blocks. Cell density gradually reduced from tissue blocks, Figure 1A. Four to 6 days later, cell confluency reached 80%‐90% and a swirl colony growth form was represented, Figure 1B. Cells after the first passage were used to detect their surface markers by flow cytometry. The results suggested that CD105 and CD90 were strongly expressed, Figure 1D‐E, whereas CD45 and CD34, the specific markers of hematopoietic cell surface, were hardly expressed, Figure 1F‐G. Figure 1C was served as isotype control.

To evaluate the differentiation abilities of hUCMSCs, the confluent cells at passages 2‐4 were cultured in differentiation mediums for induction of adipocyte‐ and osteocyte‐like cells. Cultured in adipogenic medium, hUCMSCs formed lipid droplets in cytoplasm, which were stained with Oil Red O, Figure 1H. hUCMSCs cultured in osteogenic differentiation medium showed the characteristics of osteoid formation as shown by Alizarin Red S staining, Figure 1I.

### 3.2 Effects of hUCMSCs on the peritoneal function changes

In Group MGO, UF was significantly decreased comparing that in Group Control (*P *<* *.05). Ultrafiltration volume in Group MGO + hUCMSCs, which was treated with hUCMSCs, tended to be increased but had no significant changes observed comparing that in Group MGO (*P *>* *.05). D/P in Group MGO was significantly increased comparing that in Group Control (*P *<* *.05). Treated with hUCMSCs, D/P in Group MGO + hUCMSCs was tended to be decreased comparing that in Group MGO but not significant (*P *>* *.05). D2/D0 in Group MGO was significantly decreased comparing that in Group Control (*P *<* *.05). After hUCMSCs treatment, D2/D0 in Group MGO + hUCMSCs was significantly increased comparing that in Group MGO (*P* < .05), seen in Table 1.

**Table 1:** Effects of hUCMSCs on the peritoneal function changes

	Group control	Group MGO	Group MGO + hUCMSCs
UF (mL)	10.20 ± 2.30	5.08 ± 1.56^a^	6.00 ± 2.41
D/P	0.54 ± 0.12	0.73 ± 0.09^a^	0.62 ± 0.09
D2/D0	0.70 ± 0.06	0.48 ± 0.08^a^	0.61 ± 0.12^b^

UF, Ultrafiltration volume; P, plasma creatinine concentration; D, dialysate creatinine concentration; D2, dialysate fluid glucose concentration; D0, peritoneal dialysate fluid glucose concentration.

a *P *< .05 vs group control.

b *P *< .05 vs group MGO + hUCMSCs.

### 3.3 Effects of hUCMSCs on the peritoneal histological changes

**Figure 2:**
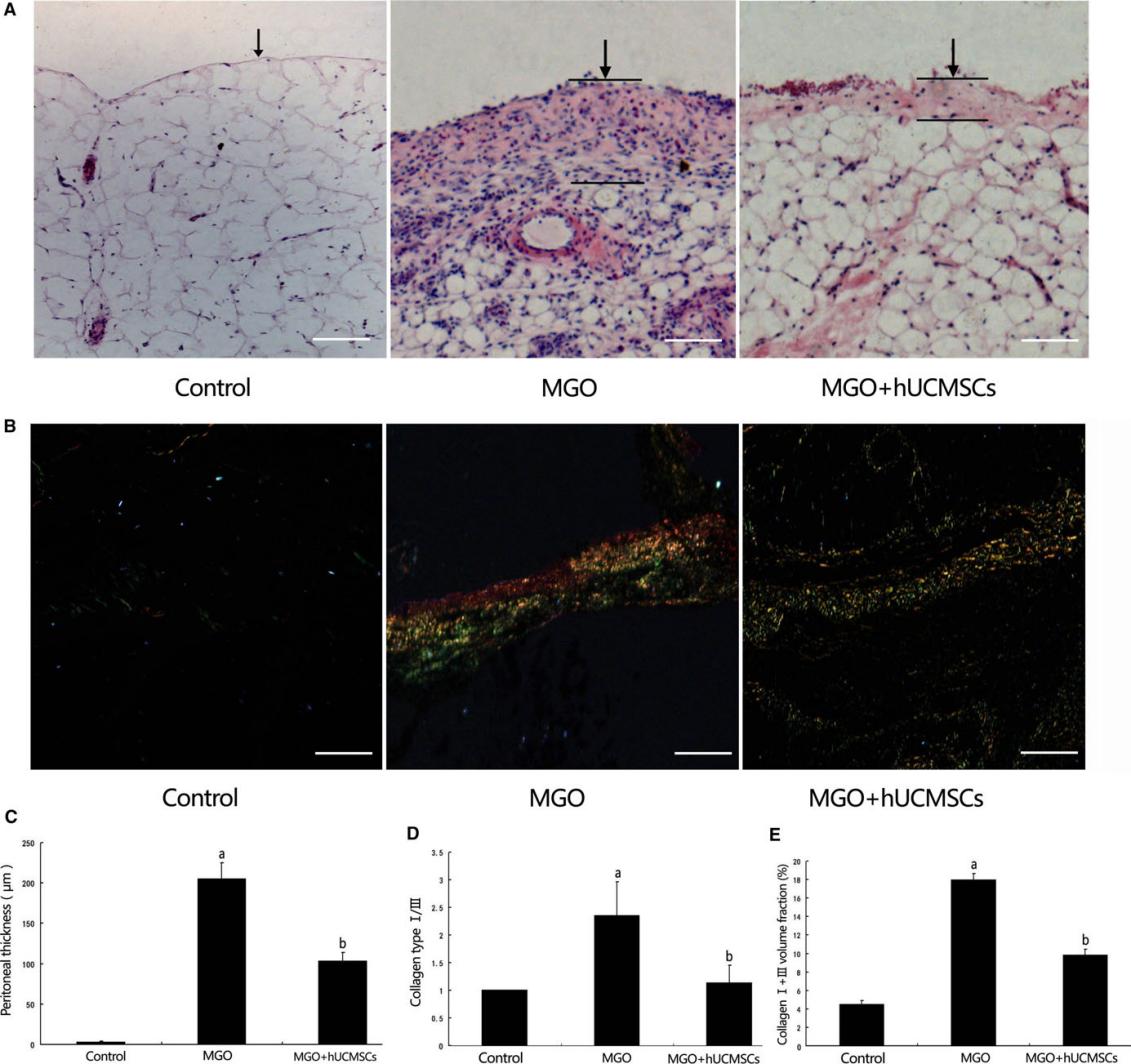
The effects of hUCMSCs on peritoneal thickness and collagen expression in MGO‐induced peritoneal damage. A, Histological findings of peritoneum stained with haematoxylin and eosin. B, Peritoneal fibrotic areas stained with Sirius Red; C, Thickness of the peritoneum was analysed in Group Control, Group MGO and Group MGO + hUCMSCs; D, Collagen type I/III depicted in (C) was analysed in Group Control, Group MGO and Group MGO + hUCMSCs; E, Collagen type I+III volume was quantified in Group Control, Group MGO and Group MGO + hUCMSCs (n =* *10‐12). Data were analysed by Student's *t* test and displayed as mean ± SEM of 10‐12 rats per group. ^a^ *P* < .05 vs Group Control, ^b^ *P *< .05 vs Group MGO + hUCMSCs (×400 magnification, bar = 50 μm)

In Group MGO, thickness of the peritoneum was substantially increased compared that in Group Control, in which submesothelial fibrotic tissue was scarce (205.2 ± 20.1 μm vs 3.7 ± 0.4 μm, *P *<* *.05). After administration of hUCMSCs, peritoneal samples showed significant decrease in peritoneal thickness (103.5 ± 10.3 μm) comparing to MGO‐induced rats (*P *< .05), Figure 2A, C‐E.

Comparing with Group Control, we found that the collagen type I/III ratio was significantly increased in Group MGO (*P *<* *.05) by Sirius Red staining. Administration of hUCMSCs significantly decreased the collagen type I/III ratio (*P* < .05), Figure 2B.

### 3.4 Effects of hUCMSCs on EMT in the MGO‐induced peritoneal tissues in rats

**Figure 3:**
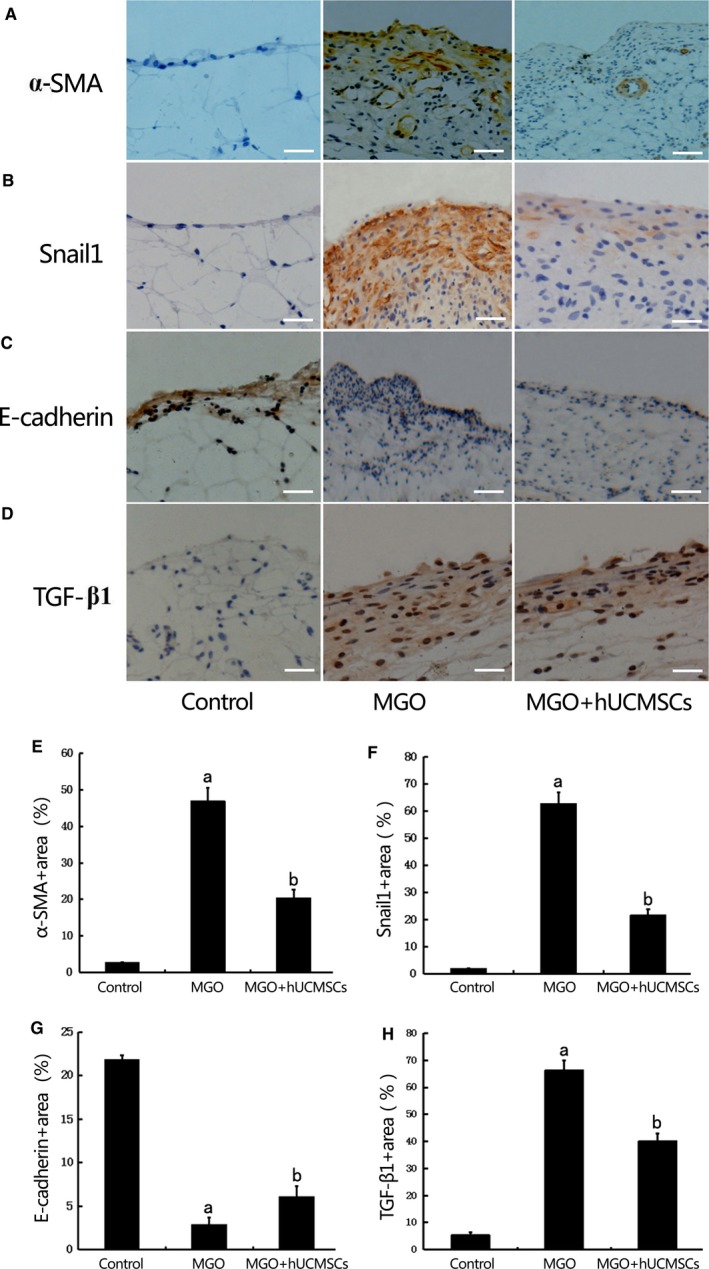
Effects of hUCMSCs on epithelial to mesenchymal transition (EMT) in the MGO‐induced peritoneal tissues in rats. The expression of α‐SMA (A), Snail1 (B), E‐cadherin (C) and TGF‐β1 (D) were detected by immunohistochemistry in peritoneal tissues of Group Control, Group MGO and Group MGO + hUCMSCs (×400 magnification, bar = 100 μm).(E‐H)Quantitative analysis revealed that α‐SMA, Snail1 and TGF‐β1 protein expression were significantly higher, and E‐cadherin was significantly lower in rats treated with MGO than that in control rats. hUCMSCs significantly down‐regulated α‐SMA, Snail1 and TGF‐β1 and up‐regulated E‐cadherin expression in MGO‐induced peritoneal fibrosis (PF) rats(n* *=* *10‐12). Data were analysed by Student's *t* test and displayed as mean±SEM of 10‐12 rats per group. ^a^*P*<0.05 vs Group Control, ^b^*P*<0.05 vs Group MGO

The effects of hUCMSCs on peritoneal EMT were explored in MGO‐induced PF rats. After injection of MGO, the immunohistochemical staining of the peritoneum revealed that α‐SMA and Snail1 expression were found in the upper layer of the submesothelial compact zone on week 2, as well as in vascular smooth muscle cells (Figure 3A‐B). Treatment with hUCMSCs significantly suppressed the extent of the α‐SMA and Snail1‐positive area on week 2 comparing with the rats receiving the vehicle (Figure 3E‐F).

E‐cadherin was significantly lower in rats treated with 20 mmol/L MGO than control, and hUCMSCs significantly attenuated EMT in MGO‐induced PF rats (Figure 3C and G). Meanwhile, we also found that EMT inducer TGF‐β1 expression was remarkedly increased in Group MGO comparing with that in Group Control and hUCMSCs significantly suppressed TGF‐β1 expression in Group MGO + hUCMSCs (Figure 3D and H).

### 3.5 MiRNA expression profiles associated with EMT

**Figure 4:**
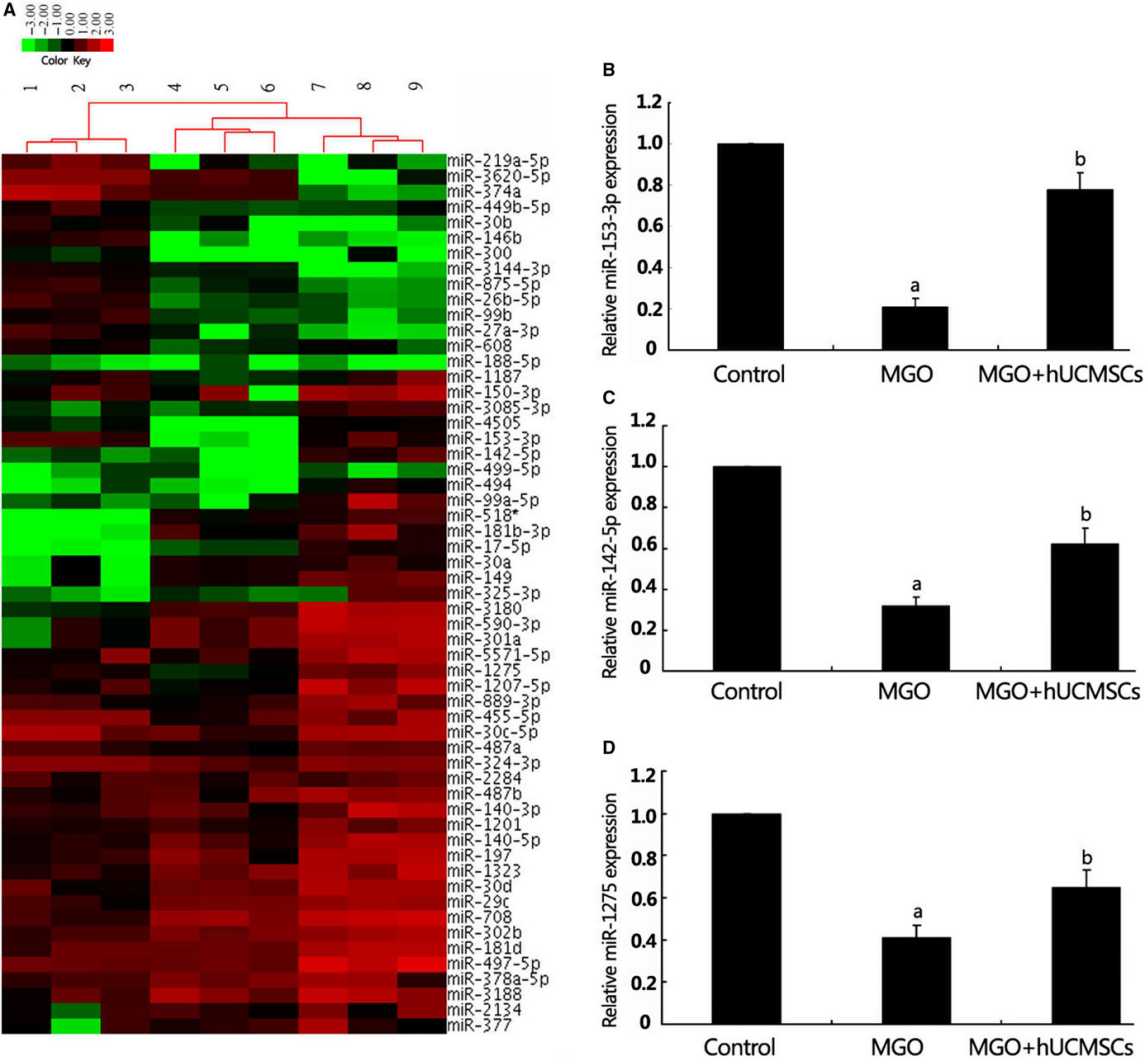
Analysis of miRNA expression profiles associated with epithelial to mesenchymal transition (EMT). A, a hierarchical clustering heat map of miRNA expression profile was obtained using fold change in miRNA expression. The analysis categorized three clusters corresponding to Group Control, Group MGO and Group MGO + hUCMSCs. The method of complete linkage was used as a clustering method. B‐D, the expression of miR‐153‐3p (B), miR‐142‐5p (C) and miR‐1275 (D) was significantly decreased in Group MGO relative to Group Control and up‐regulated in Group MGO + hUCMSCs. ^a^ *P *< .05 vs control group, ^b^ *P *< .05 vs Group MGO (n* *=* *6)

In order to highlight the effect of hUCMSCs on EMT in the MGO‐induced peritoneal tissues of rats, we sought to identify differentially expressed miRNAs related to EMT using miRNA microarrays. Among the 54 differentially expressed miRNAs in Group MGO compared to Group Control, 30 were up‐regulated and 30 were down‐regulated. After hUCMSCs treatment, 18 miRNAs were down‐regulated and 49 were up‐regulated (Figure 4A). The overlapped miRNAs down‐regulated in the MGO‐induced peritoneal tissues, and miRNAs up‐regulated after hUCMSCs treatment were selected (miR‐153‐3p, miR‐142‐5p and miR‐1275), which were further validated individually using qRT‐PCR as presented. Consistent with the miRNA array analysis, the expression of 3 overlapped miRNAs was significantly decreased in Group MGO comparing to Group Control and up‐regulated after hUCMSCs treatment (Figure 4B‐D), which is consistent with the miRNA array analysis. In both the miRNA array and the qRT‐PCR analyses, miR‐153‐3p expression demonstrated the greatest increase in hUCMSCs‐treated peritoneal tissues in Group MGO + hUCMSCs. Several previous studies demonstrated that miR‐153 played a critical role in suppressing EMT in tumours.^12^, ^13^ Therefore, miR‐153‐3p was chosen to explore the association between miRNAs and EMT during the repairing process of hUCMSCs on MGO‐induced PF rats.

### 3.6 Effects of hUCMSCs and hUCMSCs‐CM on TGF‐β1‐induced miR‐153‐3p expression in RPMCs

**Figure 5:**
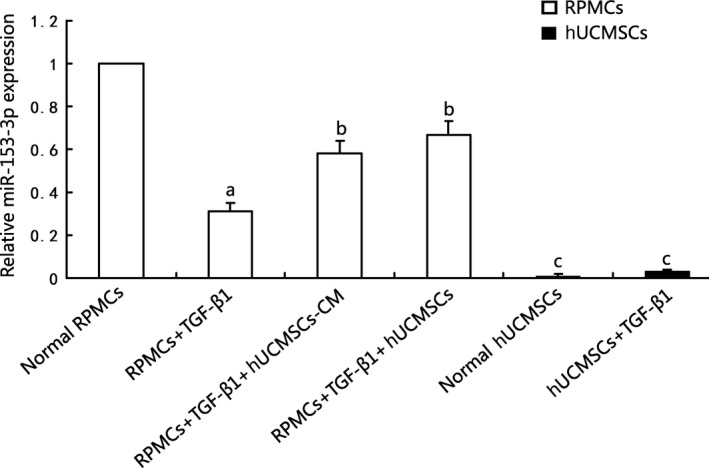
Effects of hUCMSCs and hUCMSCs‐CM on TGF‐β1‐induced miR‐153‐3p expression in RPMCs. MiR‐153‐3p was significantly down‐regulated in RPMCs stimulated with 2.5 ng/mL TGF‐β1 comparing that in normal RPMCs (*P *<* *.05), whereas cocultured with hUCMSCs or incubated with hUCMSCs‐CM, miR‐153‐3p expression of RPMCs was significantly up‐regulated (*P *<* *.05). hUCMSCs hardly expressed miR‐153‐3p with or without TGF‐β1 stimulation compared to normal Rat peritoneal mesothelial cells (RPMCs), (n* *=* *6)

MiR‐153‐3p was significantly down‐regulated in RPMCs stimulated with 2.5 ng/mL TGF‐β1 comparing that in normal RPMCs (*P *<* *.05), whereas cocultured with hUCMSCs or incubated with hUCMSCs‐CM, miR‐153‐3p expression of RPMCs was significantly up‐regulated (*P *<* *.05). However, hUCMSCs hardly expressed miR‐153‐3p with or without TGF‐β1 stimulation comparing that with normal RPMCs (Figure 5). These data implied that miR‐153‐3p contributed to anti‐EMT effects of hUCMSCs treatment in RPMCs.

### 3.7 Effects of hUCMSCs and hUCMSCs‐CM on TGF‐β1‐induced EMT in RPMCs

**Figure 6:**
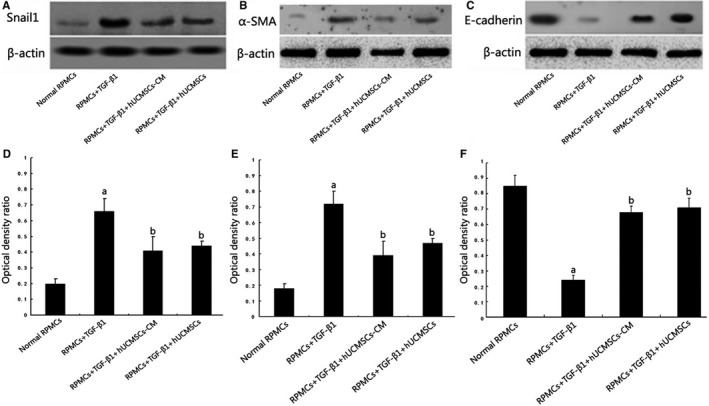
Effects of hUCMSCs and hUCMSCs‐CM on TGF‐β1‐induced epithelial to mesenchymal transition (EMT) in RPMCs assessed by Western blot and real‐time PCR assays. A‐C, Expression changes of Snail1, α‐SMA and E‐cadherin. β‐actin served as an internal control. D‐F, The bar graph shows the densitometric quantification of Snail1, α‐SMA and E‐cadherin. Stimulation of RPMCs with 2.5 ng/mL TGF‐β1 up‐regulated α‐SMA and Snail1 expression and down‐regulated E‐cadherin expression as compared that in quiescent cells (both *P *<* *.05), which was inhibited by pre‐treatment with hUCMSCs‐CM and coculture with hUCMSCs (*P *<* *.05), (n = 6)

Stimulation of RPMCs with 2.5 ng/mL TGF‐β1 up‐regulated α‐SMA (Figure 6A and D) and Snail1 (Figure 6B and E) expression and down‐regulated E‐cadherin (Figure 6C and F) expression as comparing to that of quiescent cells (both *P *<* *.05), which was inhibited by pre‐treatment with hUCMSCs‐CM and coculture with hUCMSCs (*P *<* *.05).

### 3.8 3′‐UTR of Snai1 is the direct target of miR‐153‐3p

**Figure 7:**
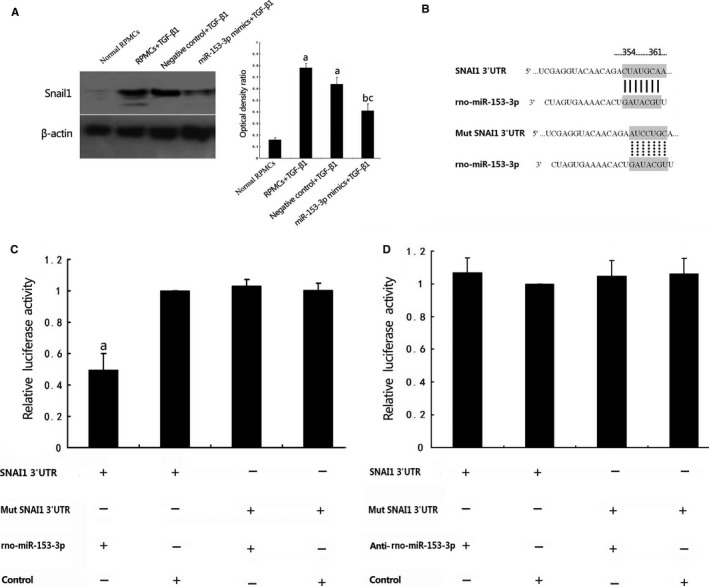
3′‐UTR of Snai1 is the direct target of miR‐153‐3p. A, RPMCs stimulated with TGF‐β1 with and without negative control molecules transfected showed significantly increased Snail1 expression assessed by Western blot. RPMCs stimulated with TGF‐β1 with miR‐153‐3p transfected showed significantly decreased Snail1 expression. β‐actin served as an internal control. ^a^ *P *< .05 vs control group. ^b^ *P *< .05 vs RPMCs+TGF‐β1 group. ^c^ *P *< .05 vs Negative control+TGF‐β1 group. B: A predicted miR‐153‐3p target site resides at nt 354‐361 of Snai1 3′‐UTR. C, Luciferase reporter assay indicates that miR‐153‐3p transfection could decrease luciferase activity in the Snai1 3′UTR group but not in the mutated Snai1 3′UTR group. ^a^ *P *< .05 vs control group. D, Transfection of anti‐miR‐153‐3p increased luciferase activity, and no significant difference was observed. There was also no apparent changes in the luciferase activities when the 3′UTR was mutated (n* *=* *3)

Computational microRNA target predictive database (TargetScan 7.0) and luciferase reporter assay were used to examine the relationship between miR‐153‐3p and Snai1. As shown in Figure 7, RPMCs stimulated with TGF‐β1 with and without negative control molecules transfected showed significantly increased Snail1 expression, which were assessed by Western blot. Rat peritoneal mesothelial cells stimulated with TGF‐β1 and miR‐153‐3p transfected showed significantly decreased Snail1 expression (Figure 7A). Transient cotransfection of miR‐153‐3p mimics with luciferase expression plasmids resulted in a significant repression of luciferase activity in RPMCs. However, mutations within putative binding site to Snai1 abrogated the effect of miR‐153‐3p mimics (Figure 7C). Furthermore, inhibition of endogenous miR‐153‐3p expression using antisense oligonucleotides increased luciferase activity after transfection and no significant difference was observed. There were also no apparent changes in the luciferase activities when the 3′UTR mutated (Figure 7D). The above results demonstrated that miR‐153‐3p contributed to anti‐EMT effects by directly targeting and inhibiting Snai1 via binding to its 3′‐UTR region.

## 4 DISCUSSION

Our data in the present study reveal that (i) hUCMSCs could ameliorate MGO‐induced peritoneal fibrosis in rats via prevention of EMT in RPMCs. (ii) miRNAs related to EMT are differentially expressed in the MGO‐induced peritoneal tissues of rats after hUCMSCs treatment. (iii) miR‐153‐3p showed the greatest increase in the MGO‐induced peritoneal tissues of rats after hUCMSCs treatment contributed to anti‐EMT effects by directly targeting Snai1.

Patients undergoing long‐term PD develop increased peritoneal thickness thus PF characterized by uncontrolled deposition of ECM.^14^ It has been proposed that collagen type I/III ratio is an important marker of ECM overexpression and accumulation.^15^The current data indicate that MSCs represent a promising candidate in direct antifibrotic treatment of various fibrotic diseases.^16^, ^17^ In our present study, after treatment of hUCMSCs, peritoneal thickness and type I/III collagen ratio were remarkedly decreased in MGO‐induced PF rats. Epithelial to mesenchymal transition of mesothelial cells plays an important role in the induction of fibrosis and early event during PD, which leads to PF.^18^ In our research, the effects of hUCMSCs on peritoneal EMT were observed in MGO‐induced PF rat. We demonstrated that EMT markers α‐SMA and Snail1 protein expression were significantly higher and E‐cadherin was significantly lower in rats administrated with MGO. hUCMSCs treatment significantly suppressed peritoneal EMT in MGO‐induced PF rat. To our knowledge, this is the first report that proves hUCMSCs could ameliorate MGO‐induced PF via prevention of EMT in PMCs. In the past decade, MSC function held great promise to mediate their beneficial effects by either differentiating into functional reparative cells or secreting paracrine factors.^19^ However, the mechanisms of which hUCMSCs exert their unique anti‐EMT effects in PF remain largely unknown. Previous study showed that DiR‐labelled MSCs mainly accumulated in the lungs first and gradually in the liver and spleen after their injection into peritoneum‐injured rats via tail tracked and monitoring by in vivo imaging.^20^ Therefore, in our present study, we conclude that the vast majority of hUCMSCs injected intravenously could not home to the damaged site and exert their anti‐EMT activities through a paracrine effect, which is mediated by the release of bioactive factors that modulate the action of PMCs. This property of hUCMSCs has prompted us to explore which bioactive factors participate in anti‐EMT process after hUCMSCs injection.

A large amount of evidence showed that miRNAs were heavily involved in the co‐ordination of EMT by suppressing the expression of different groups of transcription factors.^21^ Recent studies indicated that miRNAs might contribute to the process of EMT in PD dysfunction. MicroRNA‐129‐5p modulated EMT by targeting SIP1 and SOX4 during PD.^22^ MiR‐30a negatively regulated Snai1‐mediated EMT during PD in vitro and in vivo.^9^ In this study, we discovered that, after hUCMSCs treatment in PF rats, miR‐153‐3p was significantly increased accompanied by decreased EMT markers α‐SMA and Snail1 and increased E‐cadherin. In vitro study showed that miR‐153‐3p directly targeted and inhibited Snai1 by binding to its 3′‐UTR region in RPMCs, which suggested that miR‐153‐3p acted as a negative modulator during the process of EMT in PD.

TGF‐β1 is involved in EMT processes and favours the formation of PF.^23^ Hiwatashi et al^24^, ^25^ demonstrated that MSCs had a favourable effect on ECM regulation as well as suppression of TGF‐β1 signalling via down‐regulating the expression of Smad2/3 and Smad4. In pulmonary fibroblasts, TGF‐β1 could decrease miR‐153 expression, whereas overexpression of miR‐153 attenuated the pro‐fibrogenic activity of TGF‐β1.^26^ In our present study, hUCMSCs treatment could significantly inhibit TGF‐β1 expression and up‐regulate miR‐153‐3p levels in MGO‐induced PF rats. We also established an EMT model of cultured RPMCs treated with TGF‐β1 in vitro. MiR‐153‐3p was significantly down‐regulated in RPMCs stimulated with TGF‐β1 compared with normal RPMCs, whereas cocultured with hUCMSCs or incubated with hUCMSCs‐CM, miR‐153‐3p expression of RPMCs was significantly up‐regulated. It has become apparent that miR‐153‐3p is up‐regulated in RPMCs and involved in anti‐EMT process of hUCMSCs treatment by targeting Snai1. However, the subsequent data showed that hUCMSCs hardly expressed miR‐153‐3p with or without TGF‐β1 stimulation compared that in normal RPMCs. Two possibilities are as follows:(i) hUCMSCs could secrete a wide spectrum of biologically active factors, which might stimulate miR‐153‐3p production from RPMCs; (ii) Pri‐miR‐153, generated by hUCMSCs, were released into blood circulation after hUCMSCs intravenous injection and utilized in peritoneum. This might explain for the phenomenon that miR‐153‐3p which was almost not detected in hUCMSCs played an important role in antifibrosis of MGO‐induced PF rats.

Previous study showed that peritoneal mesothelial cells could excrete various cytokines and growth factors,^27^ and intraperitoneal transplantation of mesothelial cells was an effective way of attenuating peritoneal damage.^28^Foley‐Comer et al^29^ found that cultured and lavage‐derived mesothelial cells had the ability to attach to the injured serosal surface, proliferate and became incorporated into the reconstituted mesothelium. Recently, many investigators have suggested that some stem cells including hUCMSCs may have the potential to transform into the closest cell type within an in vivo environment with the help of factors released from tissues.^30^, ^31^, ^32^ Our present study showed that, after hUCMSCs administration, peritoneal damage was significantly improved in Group MGO + hUCMSCs. Such effects may be partly due to differentiation of hUCMSCs into functional peritoneal mesothelial cells which thus excrete miR‐153‐3p and play anti‐EMT effects in the peritoneum. Besides EMT, PF is also related to a chronic inflammatory status and an elevated oxidative stress status which results from chronic exposure to glucose degradation products, including MGO.^33^ Recent reports have demonstrated that the protective potential of MSCs was mediated by paracrine pathways that involved antioxidants and anti‐inflammatory agents.^34^, ^35^ Hence, in our present study, the mechanism of which hUCMSCs exert their beneficial effects might be that hUCMSCs injected intravenously secrete soluble cytokines into the blood to enhance the repair of injured peritoneum by suppressing inflammatory reactions which is independent from hUCMSCs differentiation.

Collectively, these studies suggest that miR‐153‐3p is a critical molecule in the protective effects of hUCMSCs during PD therapy. Our findings also suggest that miR‐153‐3p exerts its beneficial effect perhaps through direct targeting of Snai1. It is anticipated that overexpression of miR‐153‐3p may represent an effective therapeutic potential for PF and guide the development of therapeutic innovations in PD.

## CONFLICT OF INTEREST

None of the authors have any potential financial conflict of interest related to this manuscript.
